# Dissecting the Role of Immune Checkpoint Regulation Patterns in Tumor Microenvironment and Prognosis of Gastric Cancer

**DOI:** 10.3389/fgene.2022.853648

**Published:** 2022-04-19

**Authors:** Zili Zhen, Zhemin Shen, Peilong Sun

**Affiliations:** ^1^ Department of General Surgery, Jinshan Hospital, Fudan University, Shanghai, China; ^2^ Department of Surgery, Shanghai Medical College, Fudan University, Shanghai, China; ^3^ Center for Tumor Diagnosis and Therapy, Jinshan Hospital, Fudan University, Shanghai, China

**Keywords:** gastric cancer, immune checkpoint, tumor microenvironment, immunotherapy, prognosis

## Abstract

Many studies suggest that immune checkpoint molecules play a vital role in tumor progression and immune responses. However, the impact of the comprehensive regulation pattern of immune checkpoint molecules on immune responses, tumor microenvironment (TME) formation, and patient prognosis is poorly understood. In this study, we evaluated immune checkpoint regulation patterns in 1,174 gastric cancer (GC) samples based on 31 immune checkpoint genes (ICGs). Three distinct immune checkpoint regulation patterns with significant prognostic differences were ultimately identified. Moreover, GC patients were divided into two subgroups according to immune checkpoint score (ICscore). Patients with lower ICscore were characterized by a favorable prognosis and enhanced immune infiltration as well as an increased tumor mutation burden, non-recurrence, and microsatellite instability-high. Collectively, this study indicated that immune checkpoint regulation patterns were essential to forming the diversity of TME and a better understanding of that will contribute to assessing the characteristics of TME in GC, which intends to improve the development of immunotherapy.

## Introduction

On a global basis, gastric cancer (GC) is one of the most prevalent malignancies, ranking fifth in cancer incidence and fourth in cancer-related causes of death (GBD 2017 Stomach Cancer Collaborators, 2020). More than one million new GC patients were diagnosed in 2020, with approximately 769,000 deaths (Sung et al., 2021). Timely endoscopic resection or other radical surgery after diagnosis with early GC could effectively control or even cure the disease under certain conditions, whose 5-year survival rate exceeded 90% (Smyth et al., 2020). For advanced GC, chemotherapy and some targeted agents, such as trastuzumab for HER-2 positive tumors, contribute to the clinical efficacy (Janjigian et al., 2020; Zhu et al., 2021). However, due to the cytotoxicity of agents and the limitation of benefit from targeted drugs, the outcome of patients with advanced GC is relatively poor, with a median survival time of only about one year (Moehler et al., 2021; Nakajima et al., 2021). New and effective therapies are therefore an urgent necessity.

The advent of immunotherapy, especially the development of immune checkpoint molecules, has revolutionized traditional cancer therapies (Sharma and Allison, 2015b; a). Immune checkpoints can be activated, in which costimulatory proteins transmit signals to promote immune responses to pathogens. In contrast, inhibitory properties are the opposite, such as PD-1/PD-L1, and CTLA4 (Ohaegbulam et al., 2015; Postow et al., 2015; Liu and Zheng, 2018). Blocking the binding of PD-1 and PD-L1, and anti-CTLA4 antibodies can restore the antitumor activity of T cells and further kill tumor cells (Bagchi et al., 2021). Additional novel immunostimulatory checkpoint molecules are now progressing into clinical trials, including CD40, CD27, GITR, OX40, ICOS (Han and Vesely, 2019). These molecules can regulate the interaction of innate or adaptive immune cells, such as activation, suppression, and even apoptosis (Dostert et al., 2019; So and Ishii, 2019).

Immunotherapy has gradually become an important therapeutic option for advanced GC. In Chinese and Japanese guidelines, immunotherapy is considered the third-line treatment for unresectable, advanced metastatic GC. Nivolumab and Pembrolizumab, anti-PD-1 monoclonal antibodies, have been approved for third-line (or greater) therapy of PD-L1 positive advanced GC based on the results of large studies (Kang et al., 2017; Fuchs et al., 2018). However, there are few basic or clinical studies on immune costimulatory molecules for GC. The agonist antibodies of these molecules to activate the immune system to kill tumors may become a novel target for the treatment of advanced GC. Previous studies only focused on the function of a specific immune checkpoint molecule in GC, whereas a range of immune checkpoint molecules modulates the antitumor effect of the immune system in a highly coordinated manner. Thus, exploring the regulation impacts of multiple immune checkpoints on tumors and specific immune characteristics are instrumental in enhancing the comprehensive understanding of immune checkpoints for GC. The genomic information of 1174 GC samples was integrated to evaluate the regulation patterns of immune checkpoint genes (ICGs) and the correlation between the patterns of immune checkpoints and the characteristics of immune cell infiltration. Besides, we further quantified individual GC patients’ immune checkpoint regulation patterns.

## Materials and Methods

### Collection and Preprocessing of Gastric Cancer Gene Expression Data

The gene expression profiles and clinical characteristics of 1174 GC samples were obtained from the Cancer Genome Atlas (TCGA) database and Gene-Expression Omnibus (GEO) database, including TCGA-STAD (N = 371), GSE57303 (N = 70), GSE62254 (N = 300), and GSE84437 (N = 433). RNA-sequencing from the TCGA cohort was normalized to transcripts per kilobase million values. The ComBat algorithm in the “SVA” R package was utilized to eliminate batch effects between distinct datasets. We acquired somatic mutation data and copy number variation (CNV) of GC samples in the TCGA cohort from the UCSC Xena database.

### Unsupervised Clustering for 31 Immune Checkpoint Genes

We selected 31 ICGs detected in the five integrated GC datasets to identify different immune checkpoint regulation patterns. These 31 genes included 12 costimulatory molecules (CD27, CD28, CD40, CD40LG, ICOS, ICOSLG, TNFRSF18, TNFRSF4, TNFRSF9, TNFSF18, TNFSF4, TNFSF9), and 19 coinhibitory molecules (ADORA2A, BTLA, CD160, CD274, CD276, CD70, CD80, CD86, CTLA4, HAVCR2, KIR3DL1, LAG3, LGALS9, PDCD1, PDCD1LG2, TNFRSF14, TNFSF14, VSIR, VTCN1). Of these, CD160, CD274, CD276, CD40LG, CD70, CD80, CD86, ICOSLG, LGALS9, PDCD1LG2, TNFRSF14, TNFSF14, TNFSF18, TNFSF4, TNFSF9, VSIR, and VTCN1 were ligand, whereas ADORA2A, BTLA, CD27, CD28, CD40, CTLA4, HAVCR2, ICOS, KIR3DL1, LAG3, PDCD1, TNFRSF18, TNFRSF4, and TNFRSF9 were receptors. Gene network analysis was performed using STRING database (https://string-db.org/). The unsupervised learning, specifically the consensus clustering algorithm, was performed cluster analysis on the expression of 31 ICGs to identify different immune checkpoint regulation patterns. These GC patients were classified for further analysis based on the patterns.

### Functional Enrichment Analyses

Metascape (http://metasape.org) was utilized to analyze process and pathway enrichment of 31 ICGs, including Gene Ontology (GO), Kyoto Encyclopedia of Genes and Genomes (KEGG), Hallmark and Reactome (Zhou et al., 2019). The enrichment criteria were *p*-value < 0.01, minimum count >3, and enrichment factor >1.5. Gene set variation analysis (GSVA) was performed to probe the variation in biological processes between various immune checkpoint patterns using the “GSVA” R package. The gene sets of “c2. cp.kegg.v7.2. symbols”, “h.all.v7.5.1. symbols”, “c2. cp.reactome.v7.5.1. symbols” were utilized for GSVA analysis (Subramanian et al., 2005; Liberzon et al., 2011). GO and KEGG enrichment analyses were performed for differentially expressed ICGs annotation with the “clusterProfiler” R package. Adjusted *p*-values less than 0.05 were considered statistically significant. Reactome pathway enrichment analysis was performed on differentially expressed ICGs according to the threshold conditions of *p*-value<0.05 and false discovery rate (FDR) < 0.05 (Jassal et al., 2020).

### Estimation of Immune Cell Infiltration

We implemented the single sample gene set enrichment analysis (ssGSEA) algorithm to quantify the relative abundance of immune cell infiltration in the tumor microenvironment (TME), including 28 immune cell subtypes (Barbie et al., 2009; Charoentong et al., 2017). Also, the CIBERSORT algorithm was utilized to analyze the infiltration of immune cells (Newman et al., 2015). We performed the ESTIMATE algorithm to estimate the immune and stromal components of the GC samples (Yoshihara et al., 2013).

### Generation of Immune Checkpoint Score (ICscore) Signatures

Immune checkpoint-related genes were extracted from the intersection of differentially expressed genes (DEGs) between various immune checkpoint regulation patterns. Consistent clustering for the above genes divided GC patients into different groups for further analysis. Principal component analysis was utilized to construct immune checkpoint signatures, in which principal components (PC) one and two were served as signature scores. The superiority of this method was to focus the score on the collection with the most significant related (or anti-correlated) gene block in the collection, while reducing the contribution from genes that were not tracked with other collection members. We applied the following formula to clarify ICscore:

$$\text{I}\text{C}\text{s}\text{c}\text{o}\text{r}\text{e}=\sum \left(PC1_{i}+PC2_{i}\right),$$

where i represented the i^th^ immune checkpoint-related genes.

### Statistical Analyses

All statistical analyses were carried out in R 4.0.2. One-way ANOVA was utilized for multiple comparisons. Wilcox rank test was applied to compare variables between two groups. Kaplan–Meier (K-M) survival analysis and log-rank test were applied for survival analysis under different signatures. Spearman and distance correlation analyses were performed to analyze the correlations coefficients between the immune cell infiltration and expression of ICGs. Multivariable Cox regression model was carried out to identify the independent prognostic factors. We used the maftools package to display the mutation landscape. The RCircos in R package was utilized to plot the copy number variation landscape of 31 ICGs in 23 pairs of chromosomes. *p*-value < 0.05 indicated significant differences.

## Results

### Landscape of Genetic Variation of Immune Checkpoint Genes in Gastric Cancer

**FIGURE 1:**
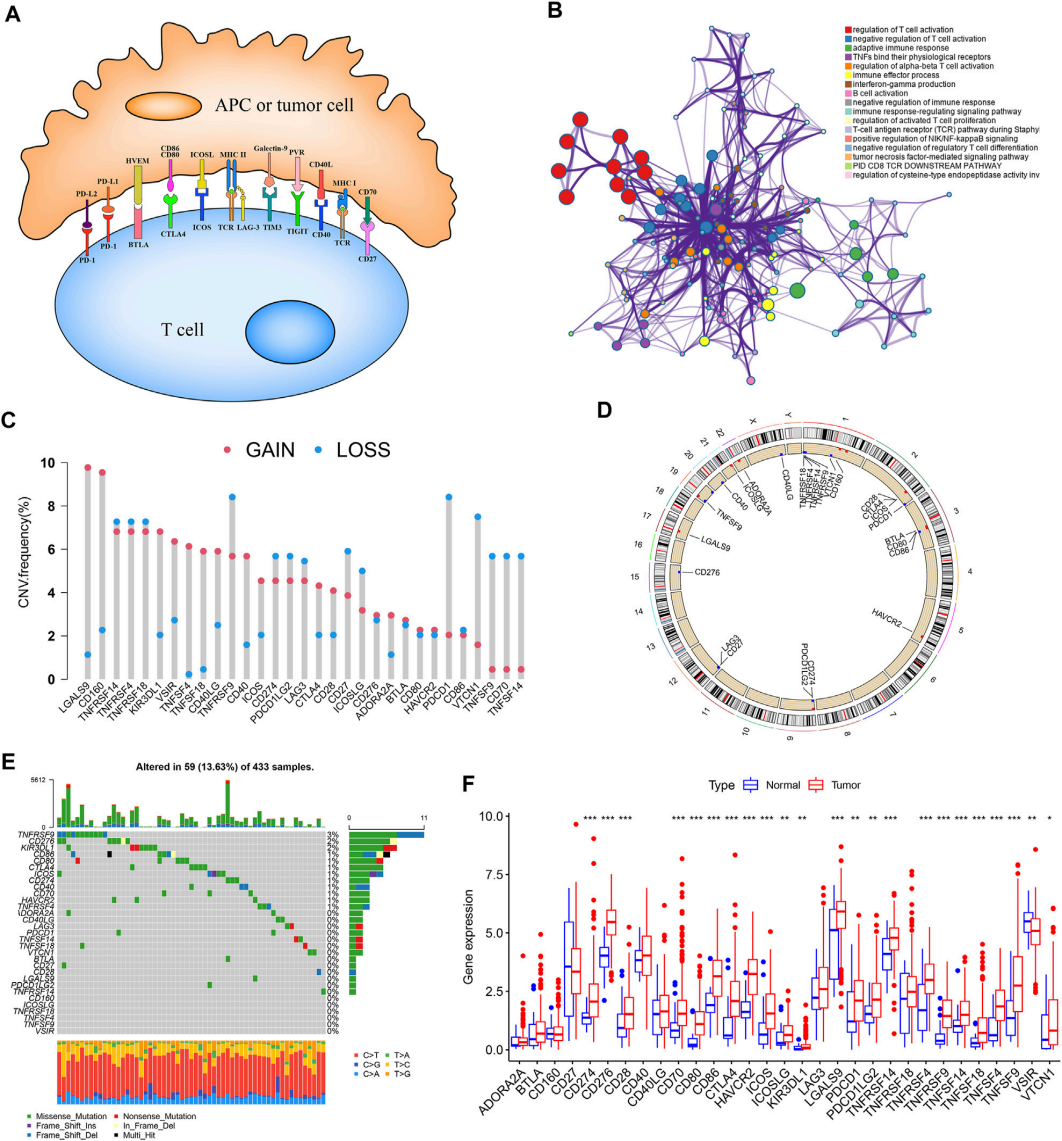
Landscape of genetic and expression variation of ICGs in GC. **(A)** Interaction of several specific immune checkpoint molecules. **(B)** Potential biological function enrichment of 31 ICGs. **(C)** The CNV variation frequency of ICGs in TCGA-STAD cohort. **(D)** The location of CNV alteration of ICGs on 23 chromosomes in TCGA-STAD cohort. **(E)** The mutation frequency of 31 ICGs in GC patients from TCGA-STAD cohort. **(F)** The expression of 31 ICGs between normal tissues and GC tissues. *p < 0.05, **p < 0.01, ***p < 0.001.

A total of 31 ICGs, including 17 ligands and 14 receptors, were initially explored for their role in GC in this study (Supplementary Table S1). Figure 1A presents the interaction of immune checkpoint molecules between T cells and tumor cells or antigen-presenting cells (APC). The GO enrichment analysis based on Metascape suggested that the 31 genes were enriched in the biological process of T cell activation regulation (Figure 1B, Supplementary Figure S1A). Examination of the CNV alteration suggested a prevalent CNV alteration in 31 ICGs (Figure 1C). Of these, the copy number of LGALS9, CD160, KIR3DL1, VSIR, TNFSF4, TNFSF18, CD40, and CD40LG was increased, while PDCD1, VTCN1, TNFSF9, CD70, and TNFSF14 had an overall frequency of CNV loss. The position of CNV alteration of some ICGs on the chromosome is displayed in Figure 1D. Also, we detected the frequency of somatic mutations in 31 ICGs in GC. Of 433 samples examined, 69 (13.63%) presented genetic alterations, mainly missense mutations. The highest mutation frequency was observed in TNFRSF9, followed by CD276 and KIR3DL1 (Figure 1E). Some ICGs had a significant mutation co-occurring relationship, such as CD274 and PDCD1LG2 (Supplementary Table S2). Interestingly, the mutation of TNFRSF9 with the highest mutation frequency was closely related to the expression of its ligand TNFSF9. TNFSF9 was significantly upregulated in TNFRSF9-mutant tumors compared to wild-type tumors (Supplementary Figure S1B). The expression levels of several other ICGs in the TNFRSF9-mutant and wild-type groups are quite different (Supplementary Figures S1C–E). We next performed a transcriptome comparison between GC tumor tissues and adjacent normal tissues to identify differentially expressed ICGs between these two groups. Apart from the downregulation of VSIR in tumor tissues, most ICGs were highly expressed in tumor tissues in relation to the adjacent normal controls (Figure 1F). These results revealed extensive variation in immune checkpoint molecules’ expression and genetic characteristics between GC tissues and normal tissues, suggesting that aberrant expression of ICGs plays a vital role in GC occurrence and progression.

### Construction of the Immune Checkpoint Patterns Mediated by 31 Immune Checkpoint Genes

**FIGURE 2:**
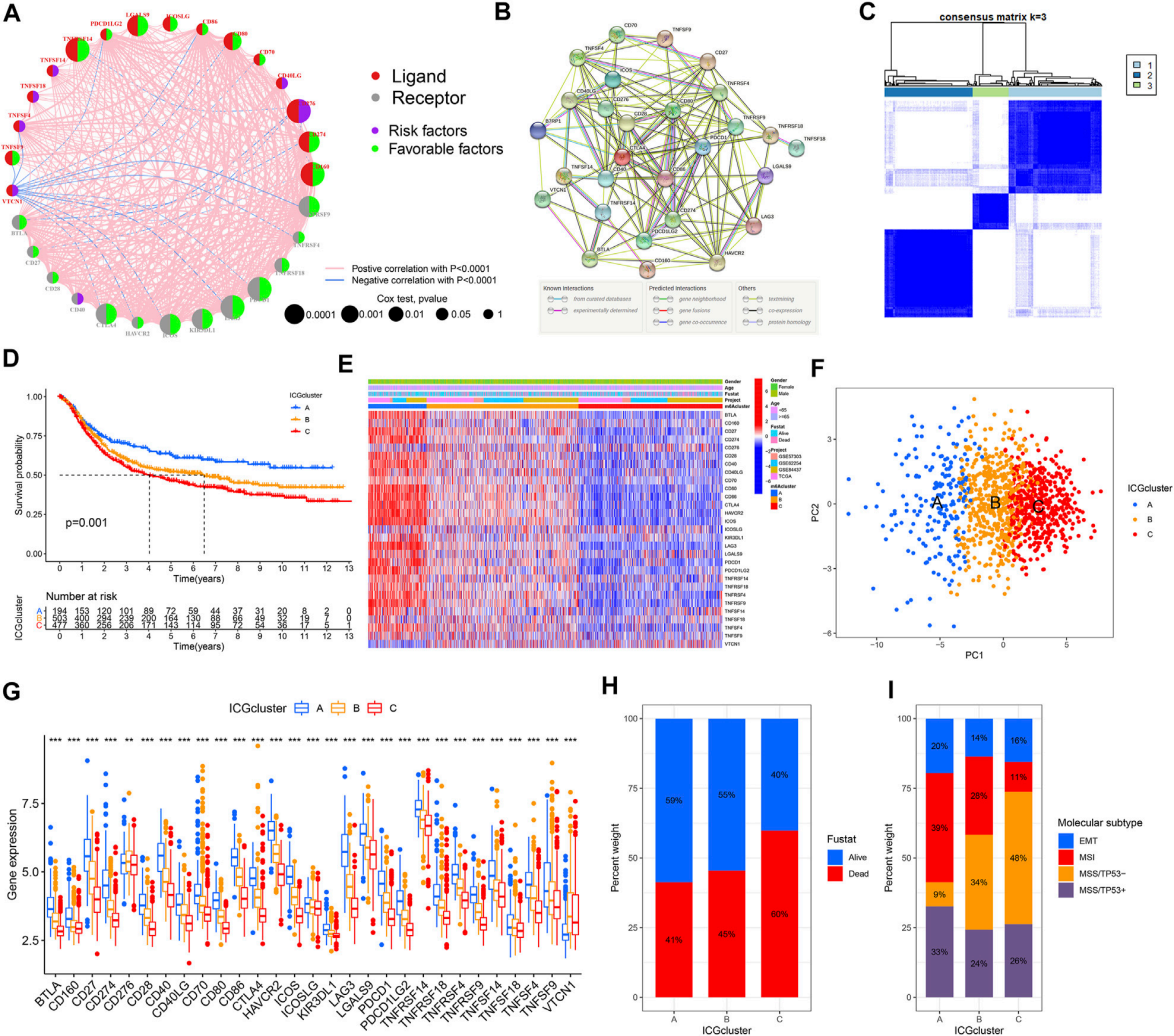
Establishment of the immune checkpoint patterns mediated by 31 ICGs. **(A)** The interaction between ICGs in GC and corresponding prognostic significance. **(B)** The protein-protein interaction network of ICGs using the STRING. **(C)** Consistent clustering matrices based on 31 ICGs for k = 3. **(D)** Survival analyses for the three immune checkpoint patterns based on 1174 GC patients. **(E)** The heatmap of 31 ICGs expression using unsupervised clustering. **(F)** Principal component analysis for the transcriptome profiles of three immune checkpoint patterns, showing a remarkable difference on transcriptome between different patterns. **(G)** The expression of 31 ICGs in three clusters. **(H)** The proportion of survival status in the three patterns. **(I)** The proportion of molecular subtypes of the GSE62254 cohort in each pattern.

Four datasets with clinical information (GSE57303, GSE62254, GSE84437, and TCGA-STAD) were integrated as a meta-cohort dataset to explore the immune checkpoint signature (Supplementary Table S3). We performed K-M analysis and univariate Cox regression analysis to clarify the prognostic values of 31 ICGs in GC patients (Supplementary Figure S2, Supplementary Table S4). A depiction of ICGs interactions and their prognostic significance for GC patients was presented in the ICGs network (Figure 2A). The interaction between immune checkpoint molecules was shown in a STRING protein–protein interaction network (Figure 2B). Significant positive associations were found among most of these ICGs, and only a few genes expression, such as VTCN1, were negatively correlated with other ICGs expressions. TNFSF14, TNFSF18, TNFSF4, CD40LG, CD276, and VTCN1 severed as poor prognostic markers, while other genes were beneficial to the prognosis.

The above results suggested that the crosstalk between various ICGs played a critical role forming various immune checkpoint patterns and was particularly relevant to the cancer progression of and immune response. Hence, we implemented consensus clustering analysis to classify samples with different immune checkpoint patterns based on the expression of ICGs (Figure 2C, Supplementary Figures S3A–I). Three different immune checkpoint patterns were determined, including 194 samples in cluster A, 503 samples in cluster B, and 477 samples in cluster C, termed ICGcluster A–C, respectively. Compared with the other three clusters, ICGcluster A exhibited an apparent survival advantage, followed by ICGcluster B, while ICGcluster C had a relatively poor prognosis (Figure 2D). There were significant differences in ICGs transcription profiles across the three immune checkpoint patterns (Figure 2E). The main characteristic of ICGcluster A was an extraordinary increase in the expression of ICGs, except for CD276 and VTCN1. The expression of ICGs in ICGcluster B was also high. Conversely, ICGcluster C manifested lower expression levels of ICGs. The unsupervised learning approach was performed to classify these samples into three classes with significant distribution differences after dimensionality reduction **(** Figure 2F). The expression of most ICGs in the three patterns showed high expression in ICGcluster A, low expression in ICGcluster C, and middle expression in ICGcluster B (Figure 2G).

Moreover, we conducted an in-depth analysis of the GSE62254 cohort to explore the characteristics of immune checkpoint patterns in different clinical features and biological behaviors. Similarly, ICGcluster A and ICGcluster B were significantly associated with prolonged survival, while ICGcluster C had a poorer survival (Supplementary Figure S3J). There were also remarkable differences in the expression of ICGs between the three patterns (Supplementary Figure S3K). Various immune checkpoint patterns could be distinguished through unsupervised learning methods (Supplementary Figure S3L). For survival status, ICGcluster C has the highest mortality rate, followed by ICGscluser B, and ICGcluster A has the lowest (Figure 2H). ICGcluster A gathered most patients with microsatellite instability (MSI) subtypes, with the least number of microsatellite stability (MSS)/TP53-subtype patients; on the contrary, MSS/TP53-subtype patients accounted for the majority of ICGcluster C, and the number of MSI subtype patients decreased sharply (Figure 2I). The above results indicated that the better prognosis of ICGcluster A patients was relevant to the immune activation caused by MSI.

### Analysis of Functional Characteristics in Distinct Immune Checkpoint Patterns

**FIGURE 3:**
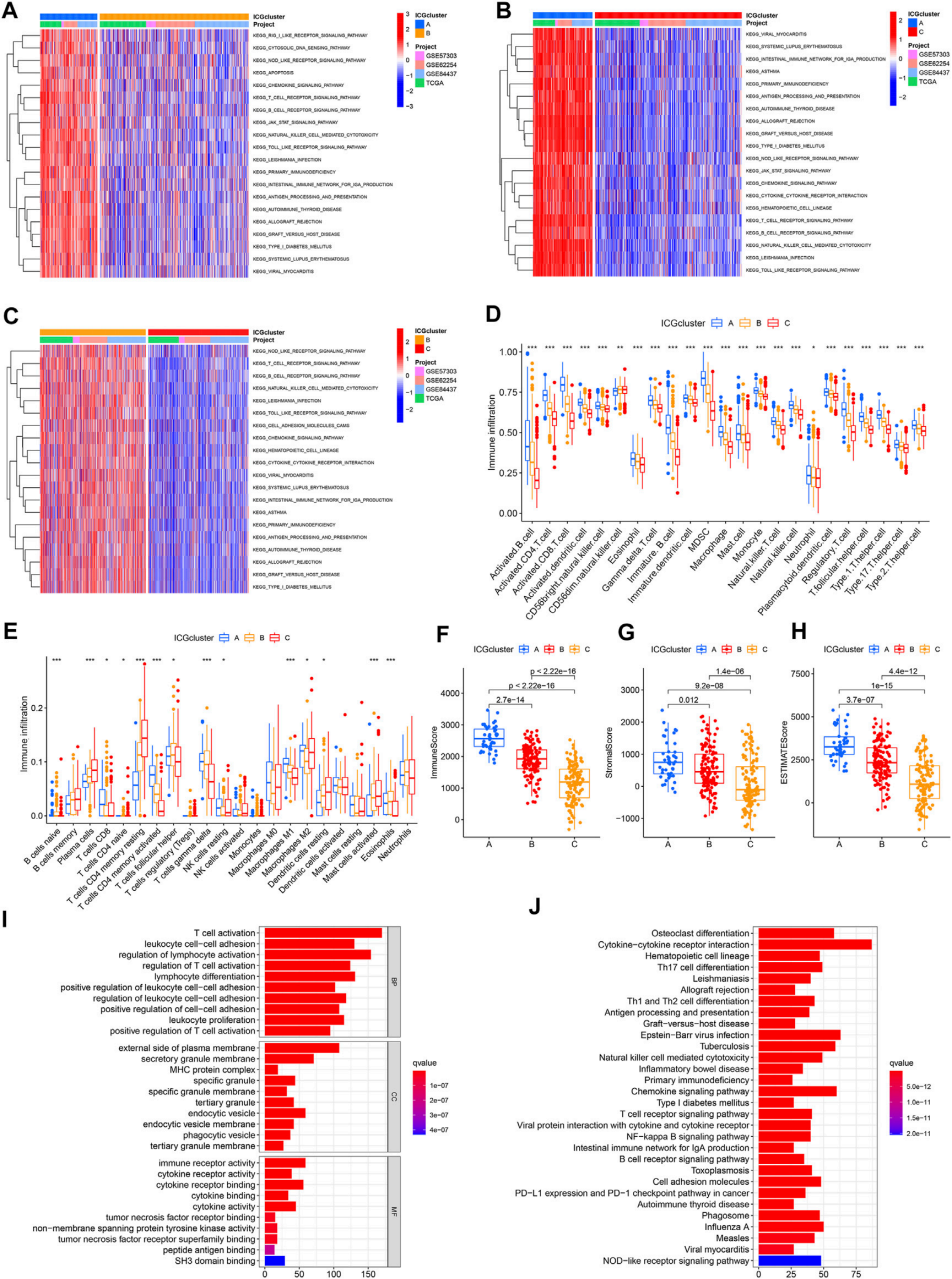
Functional enrichment analysis of three immune checkpoint regulation patterns. **(A–C)** GSVA enrichment analysis showing the activation states of biological pathways in distinct immune checkpoint regulation patterns. **(D)** The abundance of each TME infiltrating cell in three immune checkpoint regulation patterns using ssGSEA. **(E)** The abundance of immune cell infiltration of GSE62254 cohort using CIBERSORT. **(F–H)** The contents of immune cells and stromal cells in GC samples of GSE62254 cohort using ESTIMATE. **(I,J)** Functional annotation for immune checkpoint-related genes using GO and KEGG enrichment analysis.

We conducted GSVA enrichment analyses to explore the biological behaviors among these distinct immune checkpoint patterns. ICGcluster A was remarkedly enriched in immune response and activation pathways, such as T cell receptor signaling pathway, B cell receptor signaling pathway, natural killer (NK) cell mediated cytotoxicity, antigen processing and presentation, cytokine receptor interaction, NOD like receptor signaling pathway, and Toll like receptor signaling pathway, as well as apoptosis (Figures 3A,B). Likewise, ICGcluster B presented similar immune response enrichment pathways to ICGcluster C (Figure 3C). In addition, some immune-related pathways such as interleukin signaling and interferon response were considerably enriched in ICGcluster A based on Hallmarker and Reactome gene sets (Supplementary Figure S4). In contrast, the clusters with poor prognoses were prominently related to cancer metabolism and progression, including the PPAR signaling pathway. Subsequently, the analysis of immune cell infiltration under various immune checkpoint patterns suggested ICGcluster A was rich in adaptive immune cells (activated B cells, activated CD4+/CD8+ T cells, gamma delta T cells, regulatory T cells, T follicular helper cells, type 1/2/17 T helper cells) and some tumor-related innate immune cells (activated dendritic cells, NK cells, CD56 bright NK cells, CD56 dim NK cells, macrophages, MDSC) (Figure 3D). The CIBERSORT and ESTIMATE algorithm were utilized to evaluate further the immune infiltration and TME of samples from the GSE62254 cohort. Among the three patterns, T cells CD4 memory resting were more prominent in ICGcluster C, while T cells CD4 memory activating were accentuated in ICGcluster A, which could also be observed in macrophages M1. Both eosinophils and T cells gamma delta presented the lowest infiltration tendency in ICGcluster C, the opposite of plasma cells and activated giant cells (Figure 3E). Other cohorts also showed roughly the same trend (Supplementary Figure S5A–C). The evaluation of the TME suggested that the proportion of immune cells and stromal cells, as well as the ESTIMATE score, were higher in ICGcluster A than ICGcluster B, and the lowest was ICGcluster C (Figures 3F–H). However, the difference between stromal scores between ICGcluster A and ICGcluster B was much smaller than the difference between immune scores. The same situation occurred in the TCGA-STAD cohort (Supplementary Figure S5D). Notably, there was no significant difference in stromal scores between ICGcluster A and ICGcluster B in the GSE84437 and GSE57303 cohort (Supplementary Figure S5E,F). The above results indicated that ICGcluster A had the most robust immune response among the three patterns.

To further probe the potential genetic alteration and biological behaviors of various immune checkpoint patterns, we identified 1,248 immune checkpoint phenotypes related to DEGs using the limma package (Supplementary Figure S6A). GO enrichment analysis suggested that the DEGs were associated with the biological processes of lymphocyte regulation, activation, adhesion, and the molecular functions of immune receptor activation (Figure 3I). Simultaneously, according to KEGG enrichment analysis, these genes were significantly enriched in signal pathways relevant to immune response (Figure 3J). Reactome gene sets enriched in DEGs mainly including immune system (Supplementary Table S5). The results confirmed that the overlapped DEGs were characterized by immune checkpoint and immune response and could be considered immune checkpoint-related genes.

### Establishment of Immune Checkpoint Score Signatures

**FIGURE 4:**
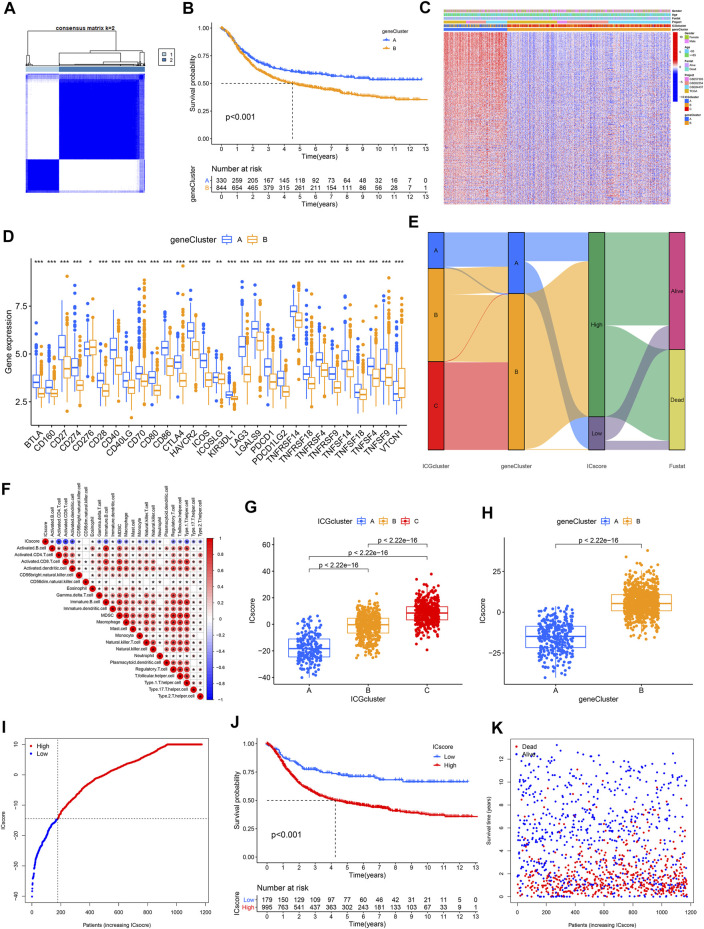
Establishment of ICscore signatures. **(A)** Consistent clustering matrices based on 1,248 immune checkpoint-related genes for k = 2. **(B)** Survival analyses for immune checkpoint gene signatures based on 1174 GC patients. **(C)** Unsupervised clustering of overlapping immune checkpoint-related genes to classify patients into two genomic subtypes. **(D)** The expression of 31 ICGs in two clusters. **(E)** Alluvial diagram showing the changes of ICGcluster, geneCluster, ICscore and survival status. **(F)** Correlations between ICscore and the TME infiltrating cell. **(G–H)** Differences in ICscore among three ICGclusters and geneClusters. **(I)** GC patients were divided into two subgroups according to the optimal cutoff value of ICscore. **(J)** Survival analyses for low and high ICscore patient groups. **(K)** Survival distribution of low and high ICscore patient groups.

We obtained two stable transcriptome phenotypes through unsupervised consensus clustering analysis of these 1,248 representative immune checkpoint-related genes, defined as geneCluster A and geneCluster B (Figure 4A, Supplementary Figure S6B–J). Survival analysis showed significant survival differences between these two immune checkpoint gene signatures in GC samples (Figure 4B). The geneCluster A was verified to be relevant to favorable prognosis, whereas geneCluster B was closely correlated with worse outcomes. The characteristic genes of immune checkpoints showed all significantly high expression in geneCluster A but were all low expressed in geneCluster B (Figure 4C). We found marked differences in ICGs expression between the two immune checkpoint gene signature subgroups, which was in agreement with the expected results of the immune checkpoint patterns (Figure 4D).

Given the heterogeneity and complexity of immune checkpoints, a scoring system that quantified the immune checkpoint regulation patterns of individual GC patients, termed as ICscore, was established on the basis of these phenotype-related genes. We used an alluvial diagram to visualize the changes in the attributes of individual patients (Figure 4E). To clarify the characteristics of the ICscore signature, we assessed its correlation with immune cell infiltration and the above two signatures. ICscore signature was negatively connected with most immune cell infiltration; the lower such a score, the more pronounced the immune checkpoint phenotype (Figure 4F). Consistently, ICGcluster A presented the lowest median ICscore compared to the other clusters, demonstrating that low ICscore could be strongly associated with immune activation-related signatures (Figure 4G). Also, geneCluster A showed a lower median score while geneCluster B had a higher median score (Figure 4H).

We further evaluated the value of ICscore in predicting the prognosis of GC patients. We divided patients into low or high ICscore groups on the basis of the best cutoff value of -14.43 (Figure 4I). Patients with lower ICscore presented better outcomes, with 5-year survival rates higher than patients with high ICGscore (71.1 vs 47.7%) (Figure 4J). Patients with higher ICscore have significantly higher mortality and shorter overall survival time (Figure 4K). Whether the ICscore could be utilized as an independent prognostic factor for GC was detected by univariate Cox and multivariate Cox regression analysis (Supplementary Figures S6K,L). In addition to age, stage, M stage, and molecular subtype, ICscore was an independent prognostic biomarker for predicting GC patients [HR 2.68 (1.83–3.93)].

### Characteristics of Immune Checkpoint Score Signatures in Tumor Mutation Burden (TMB)

**FIGURE 5:**
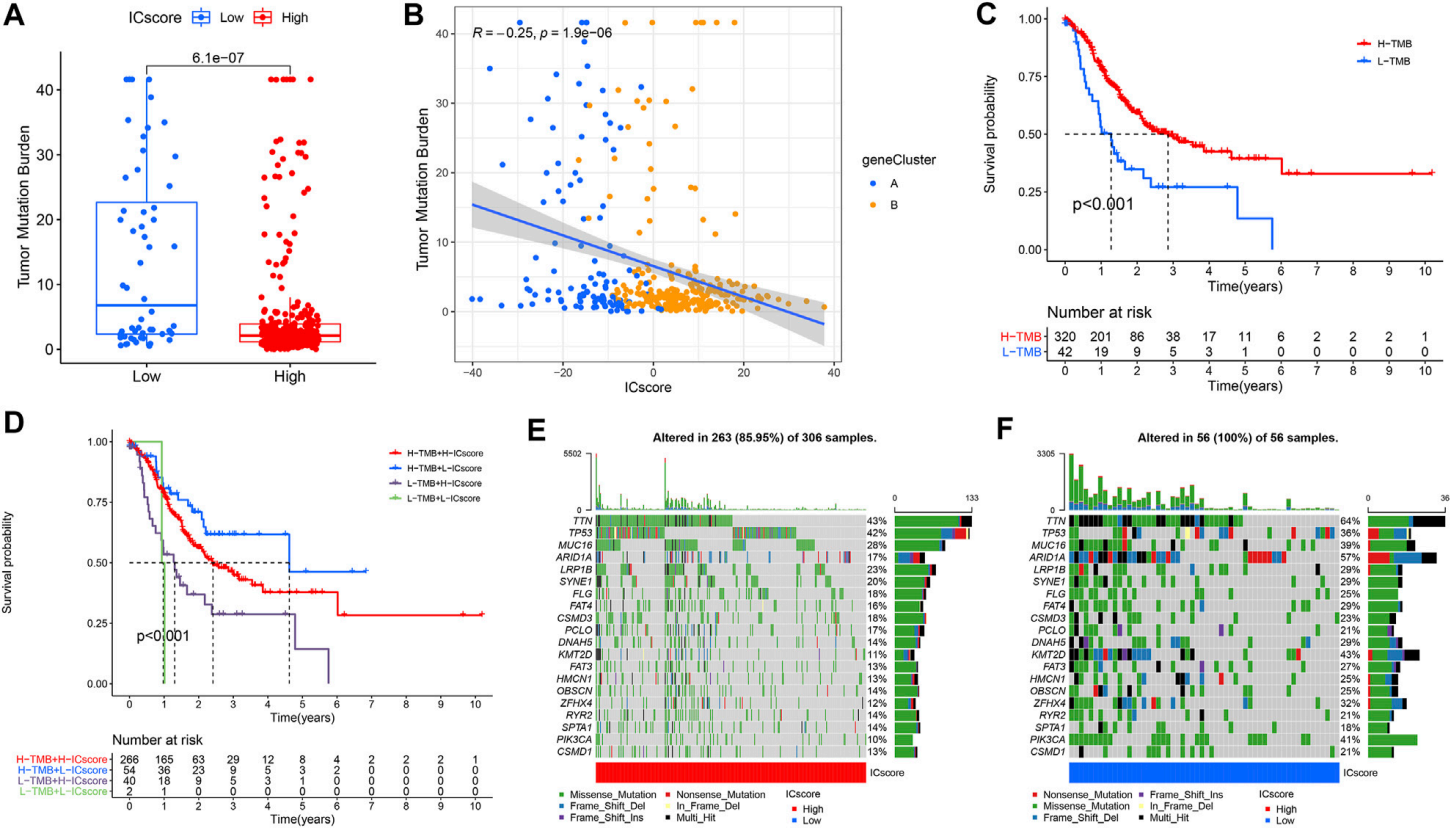
Characteristics of ICscore signatures in TMB. **(A)** Differences in TMB between high and low ICscore groups. **(B)** The relationship between ICscore and TMB. **(C)** Survival analyses for low and high TMB patient groups in TCGA-STAD cohort. **(D)** Survival analyses for patients with different degrees of TMB and ICscore. **(E,F)** The waterfall plot of TMB established by those with high ICscore and low ICscore.

The relationship between the tumor genome somatic mutations and the immune response came into increasing focus in recent years. We conducted related comparisons and found that the TMB in the low ICscore group was higher (Figure 5A). Specifically, there was an inverse correlation between ICscore and TMB; accordingly, there was a higher proportion of low TMB in geneCluster B samples (Figure 5B). After evaluating the survival of GC patients with distinct TMB, we found that the prognosis of patients with low TMB was worse than that of patients with high TMB (Figure 5C). Additionally, TMB and ICscore were assessed to evaluate the prognosis of patients. Patients with high TMB and low ICscore had a particularly favorable prognosis, far better than other groups, indicating that the increase of TMB and immune cell infiltration had a synergistic effect on improving outcomes (Figure 5D). The genomic analysis of GC patients in distinct ICscore groups suggested that genomic alterations occurred in all samples in the low ICscore group, while only 85.95% of the high ICscore group had changes (Figures 5E,F). The considerably mutated gene landscapes showed that ARID1A (57% vs 17%) and PIK3CA (41% vs 10%) had higher somatic mutation rates in the low ICscore group, while TP53 (36% vs 42%) had a higher somatic mutation rate in the high group. and the ICscore value of N3 patients was also significantly higher than N0.

### Correlation Between Immune Checkpoint Score Signatures and Clinical Characteristics

**FIGURE 6:**
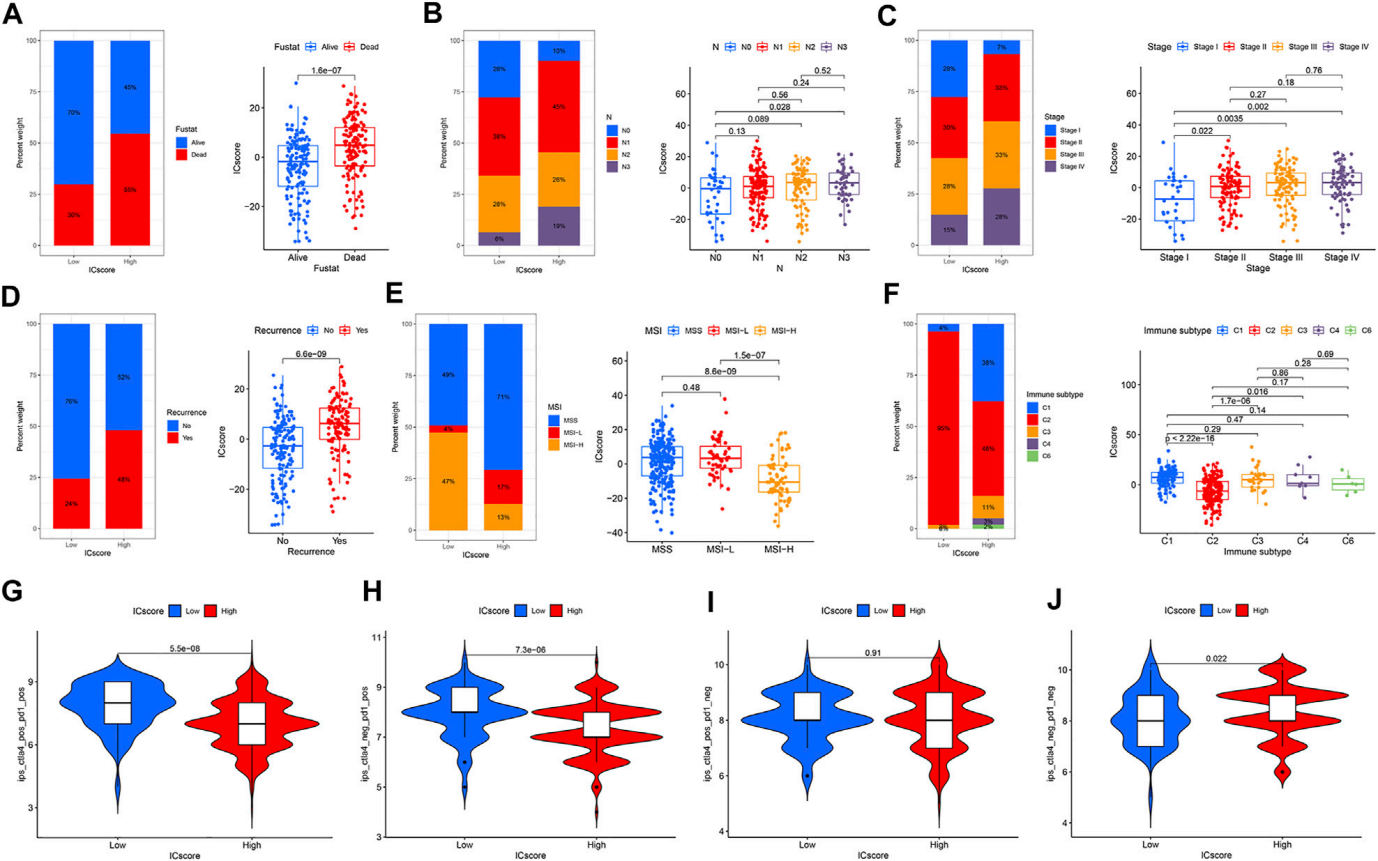
Correlation between ICscore signatures and clinical characteristics. **(A–F)** The proportions and differences of survival status **(A)**, N stage **(B)**, Stage **(C)**, recurrence **(D)**, MSI **(E)**, and immune subtype **(F)** in distinct ICscore groups **(G–J)** IPS of different ICscore groups in various CTLA4/PD-1 status.

There is complete clinical information of GC patients in TCGA cohort, including survival status, TNM stage, molecular subtypes, and immune subtypes. For patients in different ICscore groups, the mortality rate of patients in the low-risk group was strikingly lower than that of the high-risk group. Correspondingly, the ICscore value of dead patients was also markedly higher than that of alive patients (Figure 6A). The proportion of patients with lymph node metastasis in the high-risk group also increased remarkably. The ICscore value of patients with N3 was higher than patients without lymph node metastasis (Figure 6B). Considering the low-risk group, the stage of patients in the high-risk group was more aggressive, and the ICscore of stage I was lower than other stages (Figure 6C). Besides, patients in the high-risk group had a more significant proportion of relapses, and the ICscore of relapsed patients was relatively high (Figure 6D). Based on the molecular subtypes of TCGA cohort, the highly MSI subtype, characterized by better prognosis, was marked associated with lower ICscore, whereas MSI-Low and MSS had a higher ICscore (Figure 6E). In GSE62254 cohort, the ICscore of each molecular subtype was significantly different (Supplementary Figure S7). MSI subtype associated with immune checkpoint therapy had the lowest ICscore and EMT subtype related to immune privilege had the highest ICscore. In addition, the low ICscore group had the largest proportion of MSI subtype, which was helpful for immunotherapy. Previous studies divided cancers in the TCGA cohort into six immune subtypes, including Wound Healing (C1), IFN-gamma Dominant (C2), Inflammatory (C3), Lymphocyte Depleted (C4), Immunologically Quiet (C5), and TGF-beta Dominant (C6) (Thorsson et al., 2019). We observed that the low-risk group was particularly highlighted in the C2 subtype, while some C1 subtype appeared in the high-risk group (Figure 6F). The C2 subtype had the highest M1/M2 macrophage polarization and strong CD8 signal, which revealed that the better outcomes of the low-risk group were related to a robust immune response. The ICscore of C2 subtype was also lower than that of other immune subtypes. We evaluated the immunophenotypic scores (IPS) of different ICscore groups. In the case of PD-1 positive, the IPS of the low-risk group was higher, whereas there was no difference in the IPS of the two groups when PD-1 negative (Figures 6G–J). Targeted treatment of PD-1 had more incredible therapeutic benefits for patients in the low-risk group.

## Discussion

Accumulating evidence reveals that either activation or inhibition of immune checkpoints plays an essential role in antitumor efficacy. Although the function of some specific immune checkpoint molecules has been elucidated in tumor immunity, the characteristics of the overall immune response atlas mediated by immune checkpoint molecules have not yet been fully uncovered. Therefore, identifying various immune checkpoint regulation patterns in the TME will gain new insights into the tumor immunity and promotes the future precision immunotherapy.

This study determined three different immune checkpoint regulation patterns, characterized by immunophenotypes with varying degrees of anti-cancer properties. ICGcluster A was a signature with adaptive immune activation, corresponding to the immune-inflamed phenotype. In contrast, the ICGcluster C featured immunosuppression, a trend of the immune-desert phenotype (Hegde et al., 2016; Son et al., 2020). The stromal score in ICGcluster B was relatively high, suggesting the immune-excluded phenotype. The immune-inflamed phenotype, also known as the hot tumor, was manifested by a mass infiltration of activated immune cells in the TME, leading to robust immune responses (Gajewski et al., 2013; Turley et al., 2015; Chen and Mellman, 2017). Although there were sufficient immune cells in the immune-excluded phenotype, the immune cells are confined in the stroma surrounding the tumor cells instead of penetrating their parenchyma, demonstrating the incapability to kill tumor (Salmon et al., 2012; Joyce and Fearon, 2015). The immune tolerance exhibited by the immune-desert phenotype is closely related to the deficient activation of T cells (Kim and Chen, 2016). Consistent with the above discussion, there was a higher proportion of microsatellite instability in ICGcluster A, while ICGcluster C exhibited the MSS/TP53- status, indicating an unresponsive immune state (Wang et al., 2020). The characterization of immune cell infiltration in the TME in each cluster identified the reliability of our classification of distinct immune checkpoint regulation patterns.

In addition, the differences of mRNA transcriptome among these three immune checkpoint regulation patterns were related to immune-related biological pathways. The DEGs were regarded as immune checkpoint-related genes. Similar to immune checkpoint regulation patterns, three genomic subtypes were identified based on immune checkpoint-related genes. Genomic subtypes suggested that immune checkpoint regulation was necessary to establish distinct tumor immune microenvironmental landscapes. Thus, a comprehensive assessment of immune checkpoint regulation patterns could enhance our understanding of immune cell infiltration in the TME. Given the individual heterogeneity, we established a reliable and powerful ICscore system to assess the immune checkpoint regulation pattern in individual GC patients. The immune checkpoint regulation pattern characterized by the immune-desert phenotype exhibited higher ICscore, whereas patterns characterized by the immune-inflamed phenotype had lower ICscore. Furthermore, ICscore was an independent prognostic biomarker for GC. The MSI subtype, especially MSI-High, sensitive to immunotherapy, was strikingly correlated with lower ICscore (Lin et al., 2020; Yang et al., 2021). More remarkably, patients in the low ICscore group exhibited high-intensity proportions of IFN-gamma Dominant, which contributes to antitumor proliferation, inhibition of angiogenesis, and immunomodulatory effects (Mimura et al., 2018; Thorsson et al., 2019; Jorgovanovic et al., 2020; Zhen et al., 2021).

There was a notably negative correlation between ICscore and TMB. The combination of high TMB and low ICscore promoted the tumor immune response, resulting in a better prognosis for such patients. Among all molecular subtypes, the EMT subtype had the highest ICscore, suggesting a critical role for stromal activation in immune checkpoint therapy. Studies have shown that activation of the EMT pathway leaded to reduced delivery of T cells to tumor cells, thus impairing tumor-killing effects (Mak et al., 2016; Tauriello et al., 2018). Collectively, these results suggested that the immune checkpoint pattern is essential to regulate the responses of stroma and immune cells in the TME and impact the therapeutic effect of immune checkpoint therapy itself.

Some studies on signatures for predicting the prognosis of GC and evaluating TME have previously been published. Zhao et al., (2021)established a 14-gene signature to assess the overall survival of GC based on univariate Cox and LASSO Cox regression analysis of immune-related genes Although this study could predict prognosis, the main function of the 14 genes used was not to regulate immunity. Yan et al., (2020)performed ssGSEA analysis to evaluate 28 types of immune cell components of GC samples in the GEO cohorts, and identified the immune scores using LASSO Cox regression analysis The study involved only a small sample size and did not assess the relationship between scores and immune infiltration. Jiang et al., (2021)applied the CIBERSORT algorithm to evaluate immune cells and establish a tumor immune infiltration score, which was used to predict patient prognosis and chemotherapy responsiveness Relatively, our study was more targeted, starting from immune checkpoint molecules to cluster and build a signature for prognosis judgment, and eventually returning to the guidance of immunotherapy. Kim et al., (2018) identified MSI-H and EBV (+) as biomarkers of response to pembrolizumab in patients with metastatic GC Cheong et al., 2022) utilized the NTriPath algorithm to construct a GC-specific 32-gene signature for typing Molecular subtypes were associated with response to 5-fluorouracil, platinum-based chemotherapy, and immune checkpoint blockade. Our study also focused on activated immune checkpoints. Each study has a different focus and may contribute to research or clinical practice in the diagnosis and treatment of GC.

Some limitations also exist in this study. Firstly, this study only applied bioinformatics and immune infiltration algorithms to study the GC transcriptome but did not conduct basic experiments. Due to inconsistent sequencing across different GC cohorts, some molecules that may have important functions were removed during the selection process of ICGs. Therefore, our follow-up studies on immune checkpoint therapy for GC will also actively make up for the above deficiencies by using external cohorts and basic experiments.

Altogether, ICscore could be computed to comprehensively assess the immune checkpoint regulation pattern of individual GC patients and understand the corresponding immune cell infiltration characteristics of the TME, further determine the tumor immunophenotype and guide clinical practice more effectively. ICscore could serve as an independent prognostic biomarker for predicting patient survival and clinical response to immunotherapy treatments. Changes in the regulation pattern of immune checkpoints by altering immune checkpoint molecules or related genes could modify the immune cell infiltration characteristics in the TME. This strategy could enhance the immune response to GC, which contributed to developing novel immune-targeted therapeutics.

## Data Availability

The datasets presented in this study can be found in online repositories. The names of the repository/repositories and accession number(s) can be found in the article/Supplementary Material.
