# Supplementary material for: Dissecting the Role of Immune Checkpoint Regulation Patterns in Tumor Microenvironment and Prognosis of Gastric Cancer
Source: Front Genet. 2022 Apr 19;13:853648. doi: 10.3389/fgene.2022.853648 (PMC9061997; doi:10.3389/fgene.2022.853648)
Supplement: Supplementary file 2 [file DataSheet1.docx]

Supplementary Material

# Supplementary Data

None

# Supplementary Figures and Tables

## Supplementary Figures


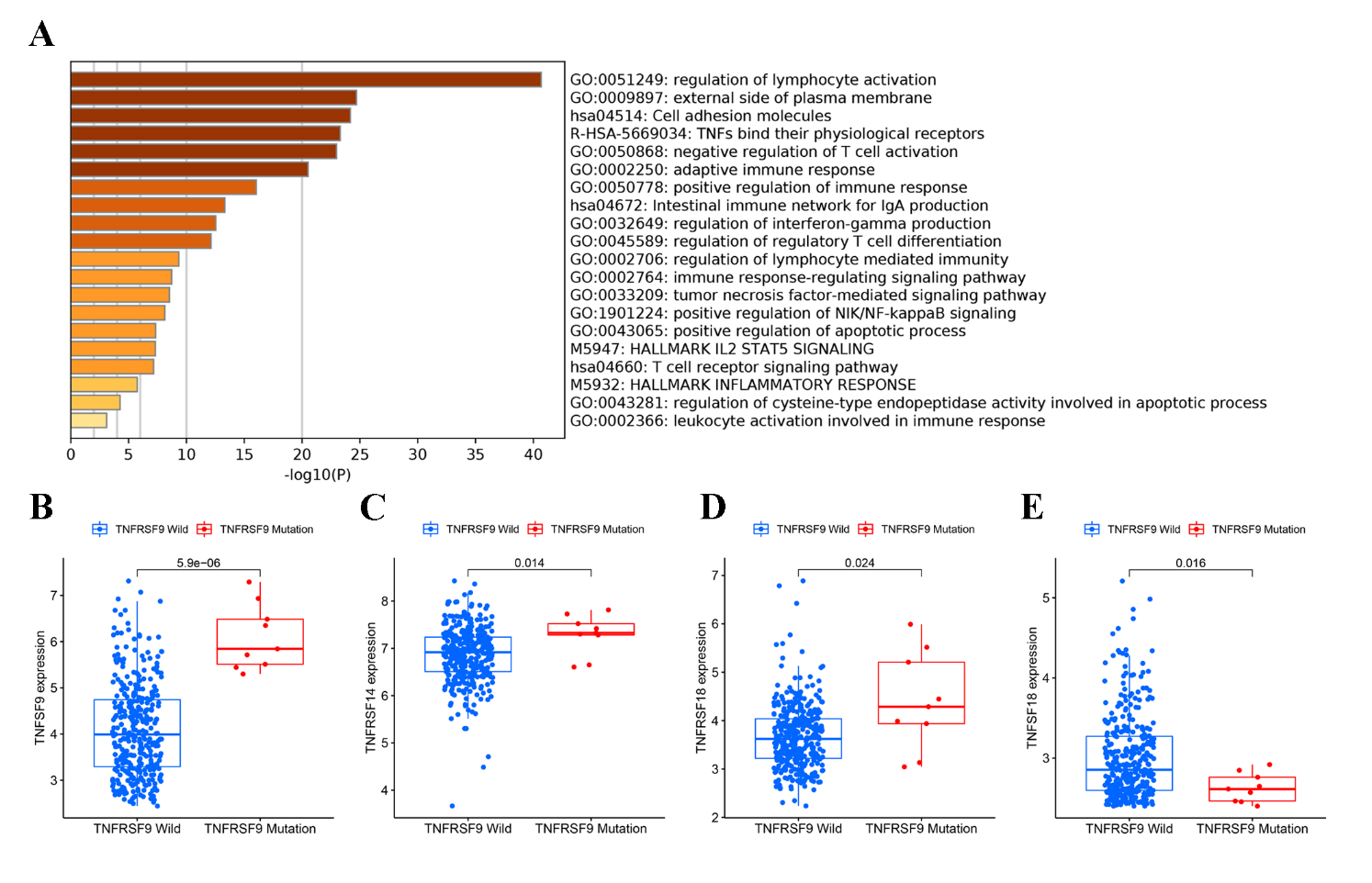
**Supplementary Figure S1.** (A) Functional enrichment analysis of 31 ICGs. (B-E) Difference in the gene expression between wild and mutation groups.


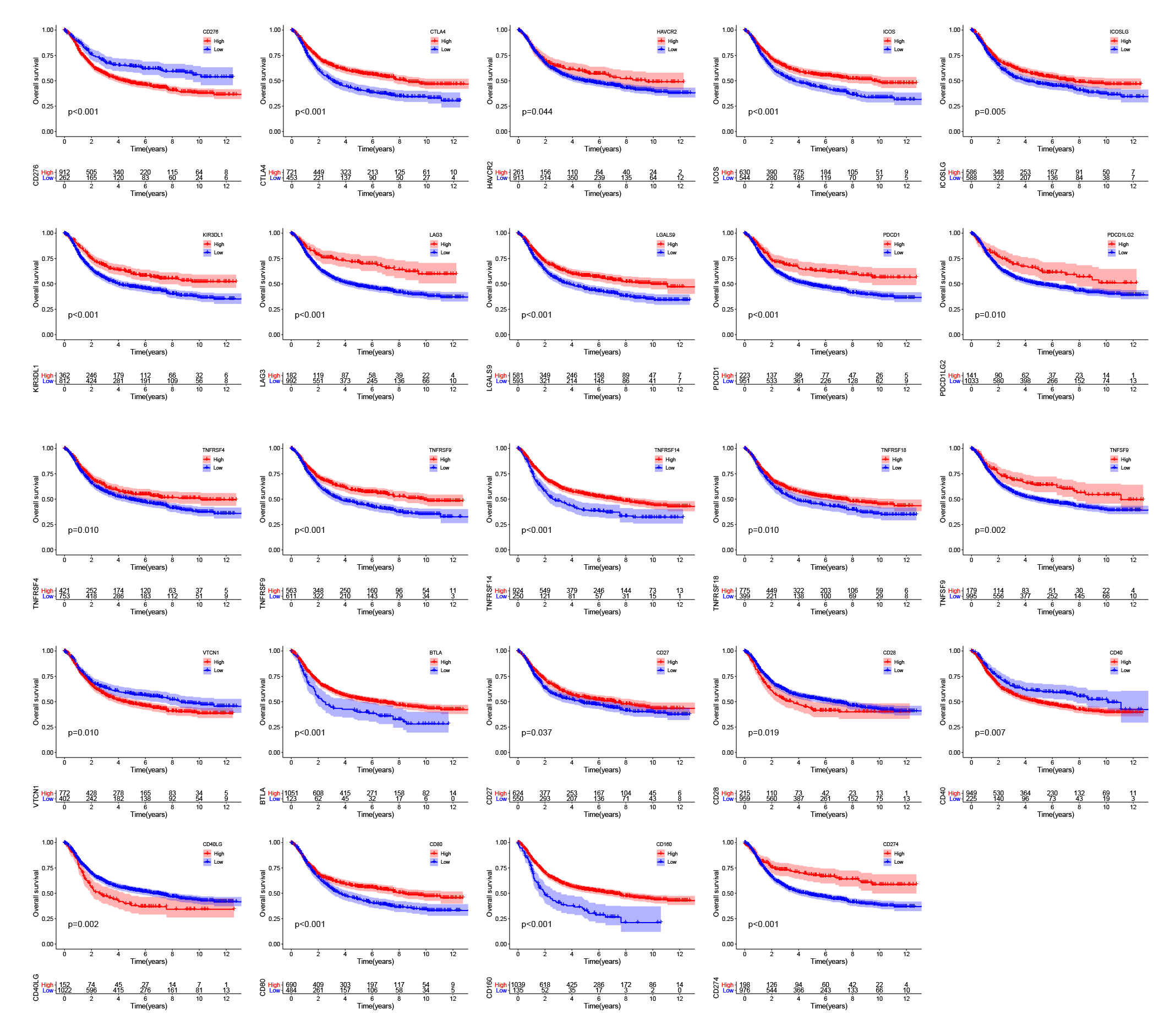
**Supplementary Figure S2.** Survival analyses for high and low expression of 31 ICGs in GC patients.


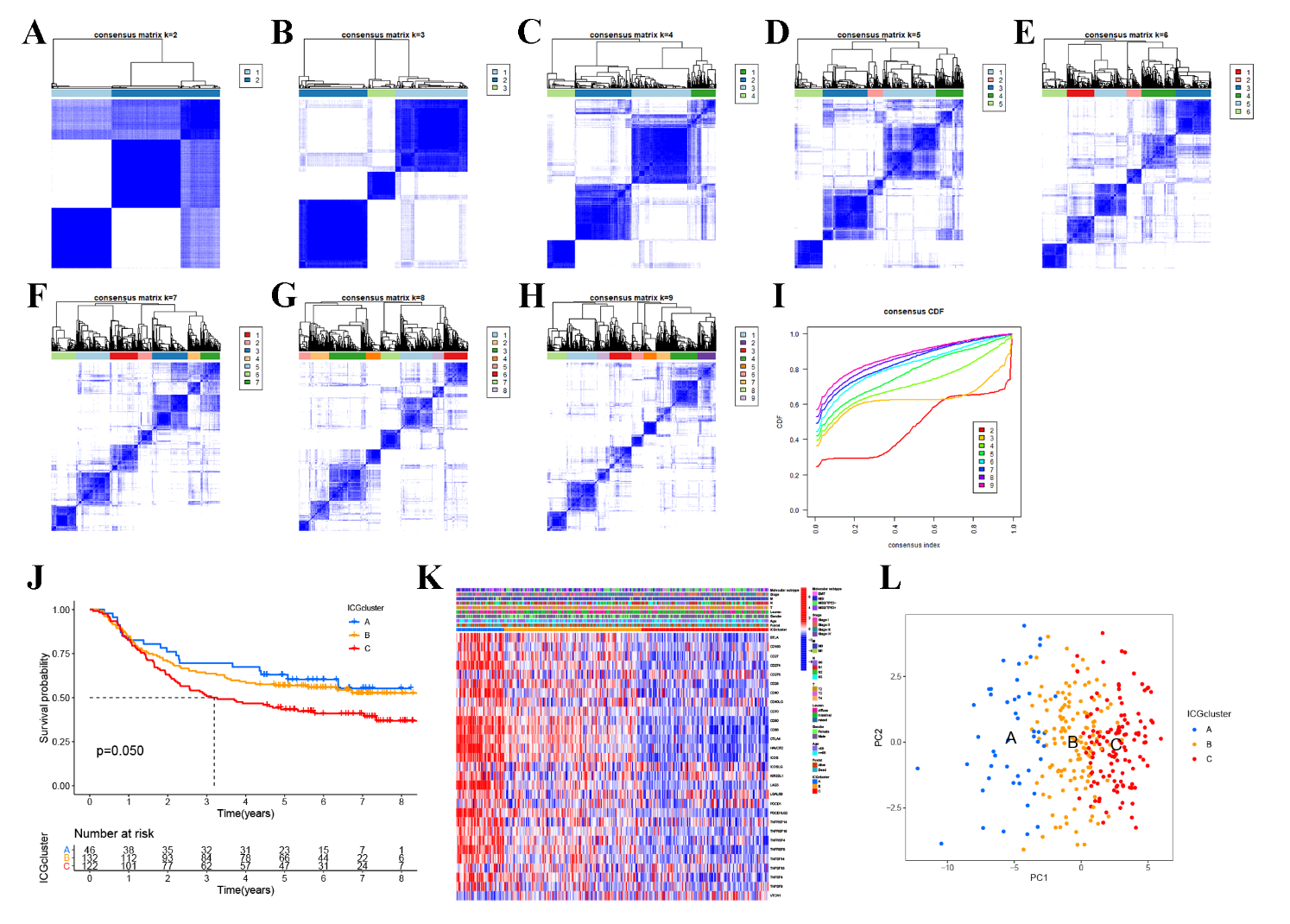
**Supplementary Figure S3.** (A-I) Consistent clustering matrices based on 31 ICGs for k=2-9. (J) Survival analyses for the three immune checkpoint patterns based on GSE62254 cohort. (K) The heatmap of 31 ICGs expression in GSE62254 cohort using unsupervised clustering. (L) Principal component analysis for the transcriptome profiles of three immune checkpoint patterns, showing a remarkable difference on transcriptome between different patterns.


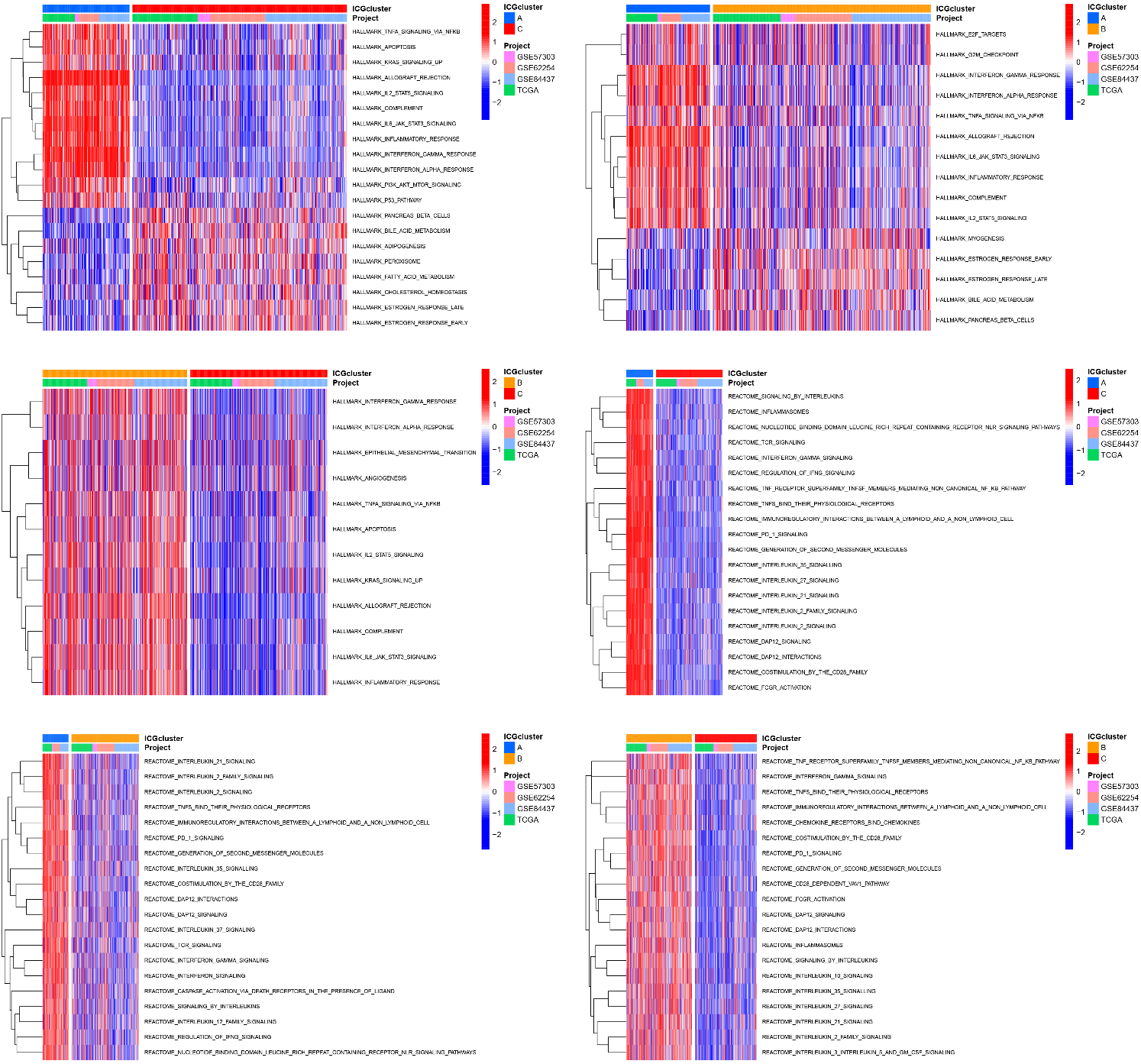
**Supplementary Figure S4.** Functional enrichment analysis of three immune checkpoint regulation patterns based on Hallmarker and Reactome gene sets.

**
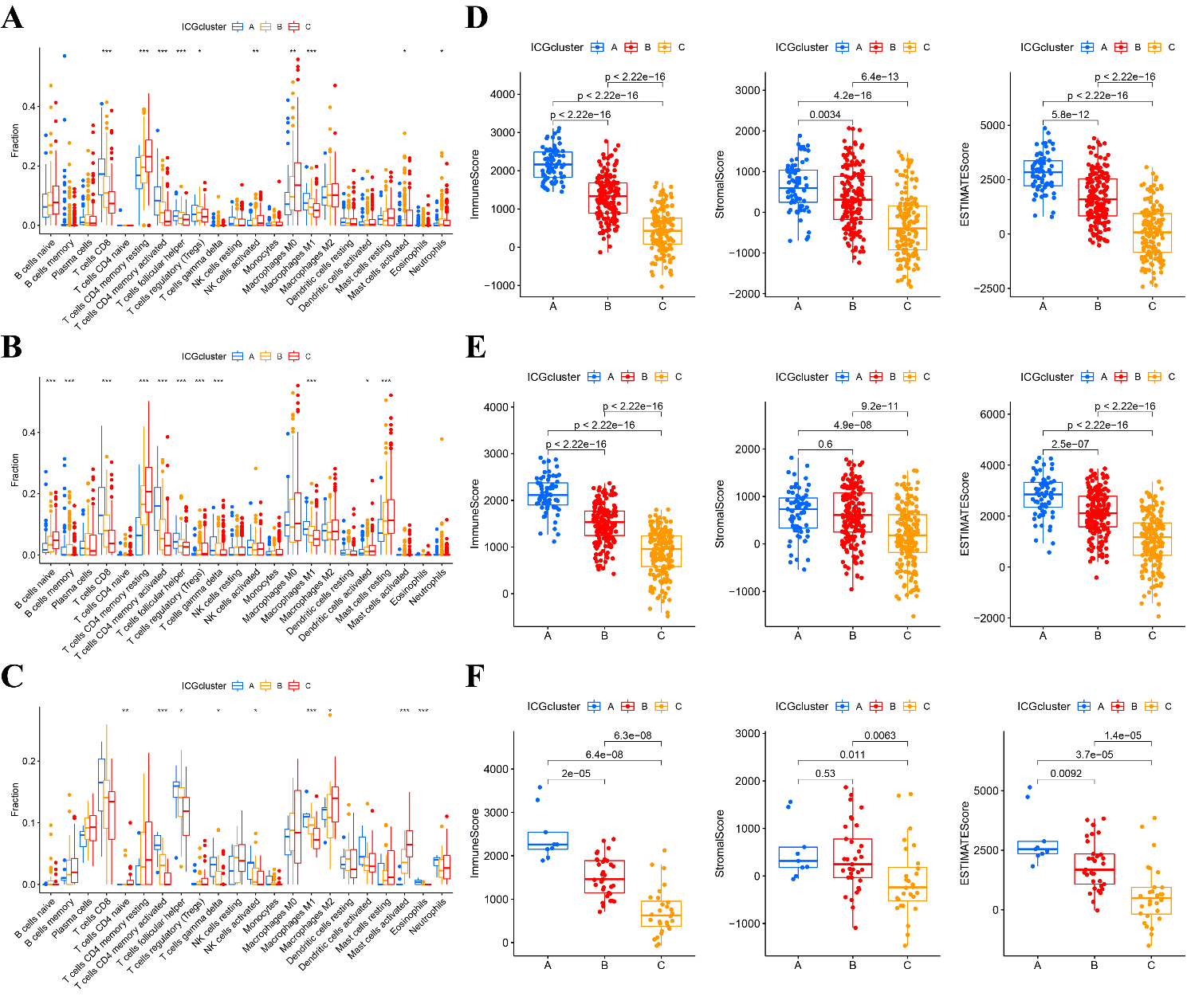
Supplementary Figure S5.** (A-C) The abundance of immune cell infiltration of TCGA-STAD, GSE84437 and GSE57303 cohort using CIBERSORT, respectively. (D-F) The contents of immune cells and stromal cells in GC samples of TCGA-STAD, GSE84437 and GSE57303 cohort using ESTIMATE, respectively.

**
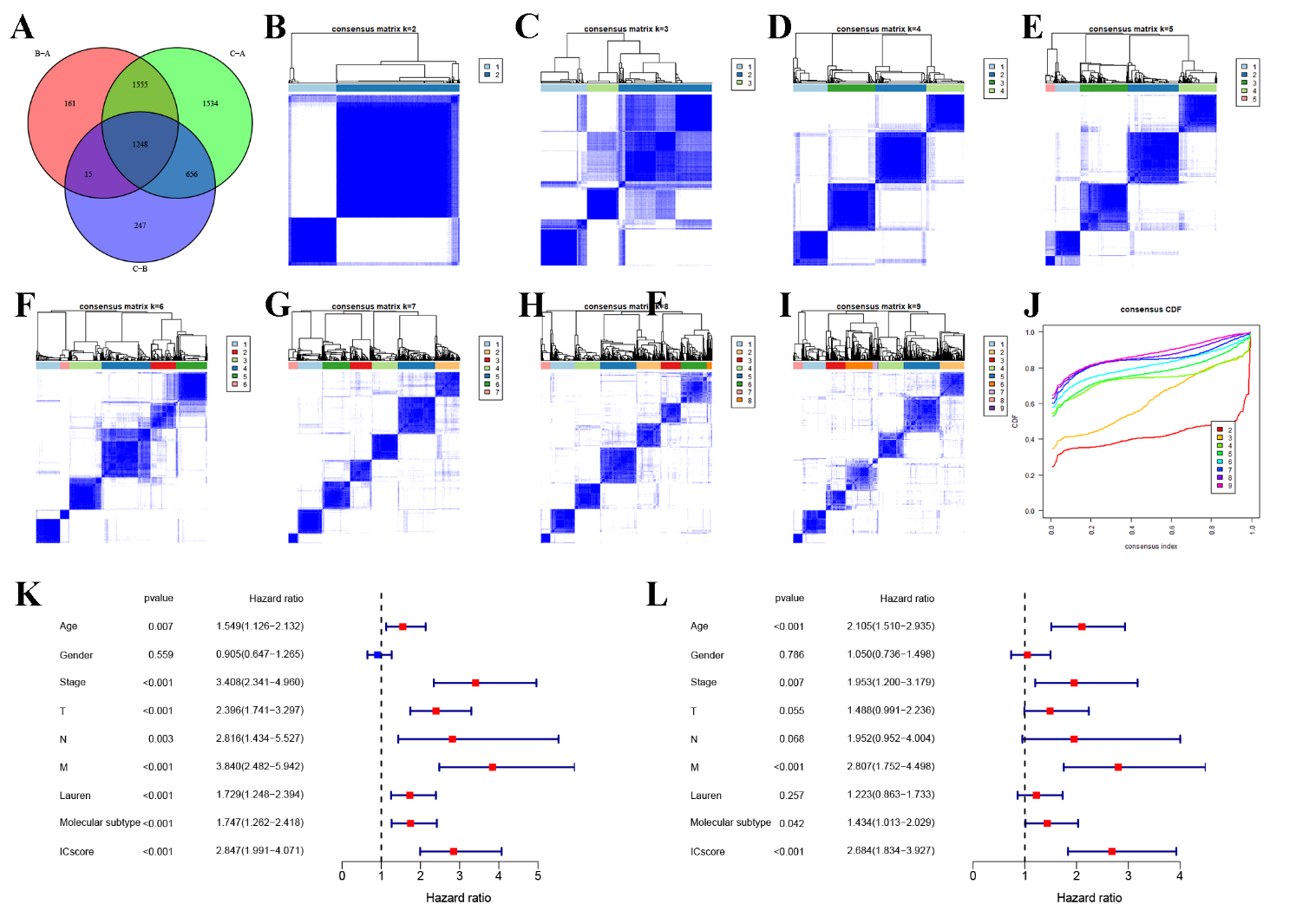
Supplementary Figure S6.** (A) 1248 immune checkpoint-related genes shown in venn diagram. (B-J) Consistent clustering matrices based on 1248 immune checkpoint-related genes for k=2-9. (K-L) ICscore could be utilized as an independent prognostic factor for GC was detected by univariate Cox and multivariate Cox regression analysis.

**
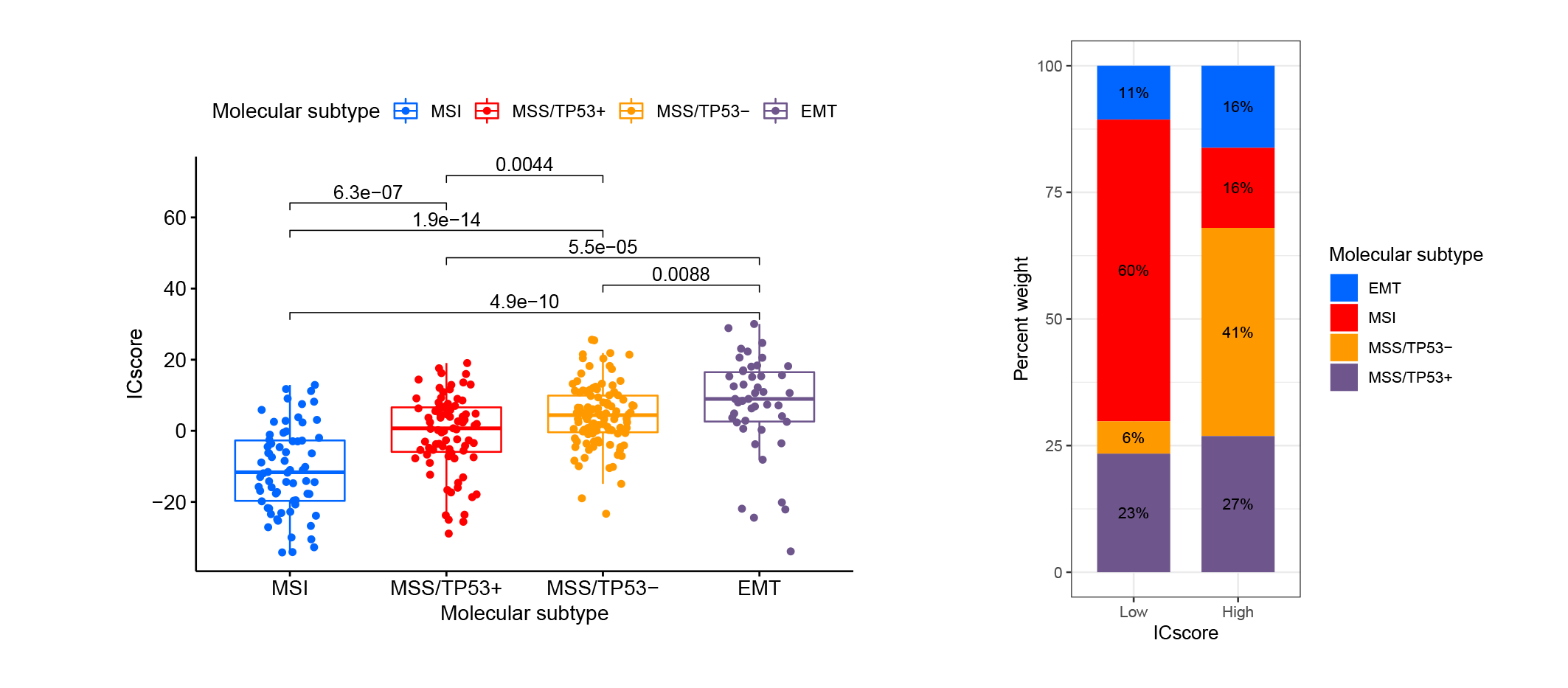
Supplementary Figure S7.** The proportions and differences of molecular subtype of GSE62254 cohort in distinct ICscore groups.

## Supplementary Tables

Supplementary Tables were available at the “Supplementary Table.xlsx”.
